# Geographic Atrophy Segmentation Using Multimodal Deep Learning

**DOI:** 10.1167/tvst.12.7.10

**Published:** 2023-07-10

**Authors:** Theodore Spaide, Jiaxiang Jiang, Jasmine Patil, Neha Anegondi, Verena Steffen, Michael G. Kawczynski, Elizabeth M. Newton, Christina Rabe, Simon S. Gao, Aaron Y. Lee, Frank G. Holz, SriniVas Sadda, Steffen Schmitz-Valckenberg, Daniela Ferrara

**Affiliations:** 1 Roche Personalized Healthcare, Genentech, Inc., South San Francisco, CA, USA; 2 Clinical Imaging Group, Genentech, Inc., South San Francisco, CA, USA; 3 Department of Electrical and Computer Engineering, University of California, Santa Barbara, Santa Barbara, CA, USA; 4 Biostatistics, Genentech, Inc., South San Francisco, CA, USA; 5 Department of Ophthalmology, University of Washington, School of Medicine, Seattle, WA, USA; 6 Department of Ophthalmology and GRADE Reading Center, University of Bonn, Bonn, Germany; 7 Doheny Eye Institute, Los Angeles, CA, USA; 8 Department of Ophthalmology, David Geffen School of Medicine at University of California, Los Angeles, Los Angeles, CA, USA; 9 John A. Moran Eye Center, University of Utah, Salt Lake City, UT, USA

**Keywords:** geographic atrophy, deep learning, segmentation, fundus autofluorescence, near infrared

## Abstract

**Purpose:**

To examine deep learning (DL)–based methods for accurate segmentation of geographic atrophy (GA) lesions using fundus autofluorescence (FAF) and near-infrared (NIR) images.

**Methods:**

This retrospective analysis utilized imaging data from study eyes of patients enrolled in Proxima A and B (NCT02479386; NCT02399072) natural history studies of GA. Two multimodal DL networks (UNet and YNet) were used to automatically segment GA lesions on FAF; segmentation accuracy was compared with annotations by experienced graders. The training data set comprised 940 image pairs (FAF and NIR) from 183 patients in Proxima B; the test data set comprised 497 image pairs from 154 patients in Proxima A. Dice coefficient scores, Bland–Altman plots, and Pearson correlation coefficient (*r*) were used to assess performance.

**Results:**

On the test set, Dice scores for the DL network to grader comparison ranged from 0.89 to 0.92 for screening visit; Dice score between graders was 0.94. GA lesion area correlations (*r*) for YNet versus grader, UNet versus grader, and between graders were 0.981, 0.959, and 0.995, respectively. Longitudinal GA lesion area enlargement correlations (*r*) for screening to 12 months (*n* = 53) were lower (0.741, 0.622, and 0.890, respectively) compared with the cross-sectional results at screening. Longitudinal correlations (*r*) from screening to 6 months (*n* = 77) were even lower (0.294, 0.248, and 0.686, respectively).

**Conclusions:**

Multimodal DL networks to segment GA lesions can produce accurate results comparable with expert graders.

**Translational Relevance:**

DL-based tools may support efficient and individualized assessment of patients with GA in clinical research and practice.

## Introduction

Geographic atrophy (GA), an advanced form of age-related macular degeneration, is estimated to affect 5 million people globally.^1^ The natural history of GA is characterized by progressive and irreversible loss of photoreceptors, retinal pigment epithelium (RPE), and choriocapillaris, leading to loss of visual function, and is highly variable.^2^ Therefore, accurate monitoring of GA progression is necessary for patient management in clinical practice when counseling patients and caregivers on the disease prognosis and in clinical research of potential new therapies as a relevant clinical parameter.^2^

Clinical trials assess GA lesion area and growth as anatomic study endpoints,^3^ with diagnosis and measurement of GA lesion area commonly assessed on fundus autofluorescence (FAF) and optical coherence tomography (OCT) as key imaging modalities. Importantly, the US Food and Drug Administration and the European Medicines Agency have accepted the use of FAF for measuring changes in GA lesion area over time as the primary endpoint in clinical trials.^2^ FAF allows demarcation of the lesions based on the absence of autofluorescence caused by the loss of lipofuscin, which harbors intrinsic fluorophores in the RPE.^2^^,^^3^ A supporting modality for visualizing GA is near-infrared reflectance (NIR), which uses longer wavelengths than FAF and is thus less affected by media opacities and signal variabilities caused by absorption of macular luteal pigments in the fovea and parafoveal area.^2^^,^^4^

The manual segmentation of GA lesions is time-consuming and subject to inter- and intragrader variabilities.^5^ RegionFinder^6^ (Heidelberg Engineering, Heidelberg, Germany), a semiautomated image-processing tool, was developed to assist expert graders in measuring the area of GA lesions from FAF images. It also allows for a more robust and reproducible documentation of grading results. Nonetheless, trained graders are still required to perform the task because user input is needed to precisely segment the GA lesion borders.^6^ Reading centers have developed the methods, training processes, and quality control measures to ensure that high reproducibility can be achieved in measuring GA from FAF images using these tools to support natural history and interventional studies.^6^

Artificial intelligence (AI)–based approaches to detect and quantify GA lesions could address a significant unmet need to precisely determine GA lesion size, enable efficient monitoring of GA progression over time, and may facilitate AI-based predictions of future GA lesion growth rates.^7^^–^^9^ Automated GA segmentation algorithms that use retinal images obtained using different modalities, including FAF,^5^^,^^10^^,^^11^ OCT,^12^^–^^14^ and NIR,^15^ as well as multimodal approaches using combinations of different imaging techniques,^16^ have been described previously.^17^ Algorithms using *k*-nearest-neighbor pixel classifiers,^5^ Fuzzy-c-means (a clustering algorithm),^7^^,^^17^ or deep convolutional neural networks (CNNs)^11^^,^^18^ led to good agreement with manual segmentation performed by trained graders. A few of these studies showed evidence of some mismatch with manually defined GA lesions, the need for high human interaction to define regions of interest, false positives, and/or missed GA lesions.^5^^,^^17^ It is important to note that although previous studies have generally focused on cross-sectional algorithm performance,^5^^,^^11^^–^^15^^,^^17^^,^^18^ longitudinal performance is more relevant to assessing endpoints in GA clinical trials.

The current study aimed to implement an end-to-end deep learning (DL) method for the automatic segmentation of GA lesions on FAF images using a multimodal approach, with both FAF and NIR images from clinical trials as inputs in two distinct end-to-end CNN-based networks; the first used a UNet architecture and the second used a method termed YNet. Importantly, in addition to cross-sectional performance in comparison to GA lesion annotations from experienced graders, the automated methods were also examined for longitudinal performance in assessing changes in GA lesions over time.

## Methods

### Data Sets and Image Processing

This retrospective study was performed using imaging data from the study eyes of patients enrolled in the Proxima A (NCT02479386; *n* = 295) and Proxima B (NCT02399072; *n* = 200) natural history studies of patients with GA.^17^ Only one eye per patient was selected as the study eye. Patients in Proxima A had bilateral GA without choroidal neovascularization (CNV) in either eye at baseline, with the total GA lesion area, ranging from 2.54 to ≤17.78 mm^2^, residing completely within the FAF imaging field 2; at least one focal lesion had to measure ≥1.27 mm^2^ in case of multifocal presentation. Proxima B had two patient cohorts: (1) GA with no CNV in the study eye and CNV in the fellow eye with or without GA (fellow eye CNV cohort), with a total lesion size of 1.27 to 17.78 mm^2^, and (2) GA with no CNV in the study eye and no CNV or GA in the fellow eye (i.e., unilateral GA [fellow eye intermediate age-related macular degeneration cohort]), with a total lesion size of 0.3 to 17.78 mm^2^, or, if multifocal, one or more focal lesions of ≥0.3 mm^2^. Full eligibility criteria for both studies have been described previously.^19^ Both studies adhered to the tenets of the Declaration of Helsinki and were Health Insurance Portability and Accountability Act compliant. The protocol was approved by the institutional review board at each site before the studies started, and all patients provided written informed consent for future medical research and analyses.

In these studies, GA diagnosis and lesion area measurements were based on fovea-centered field 2 thirty-degree FAF images captured using the Spectralis cSLO system (Heidelberg Engineering, Germany) with an automatic real-time function setting of ≥15.^19^ Corresponding NIR images captured from the same device at the same visit were used as a supportive image modality, assisting in the precise delineation of the borders of GA lesions when documentation on FAF was suboptimal (e.g., around the central foveal area).

FAF images at the screening and follow-up visits (months 6, 12, 18, and 24 and study termination) were segmented by trained human graders at the Doheny Image Reading Center (Los Angeles, CA, USA) for Proxima A and the GRADE Reading Center (Bonn, Germany) for Proxima B. At both reading centers, FAF images were longitudinally registered and lesions were delineated semiautomatically using the RegionFinder software (Heidelberg Engineering, Germany), starting by selecting a seed point and then using growth power and growth limit to adjust the segmentation algorithm of the software, with correction and constraint tools applied, as described previously.^6^ An individual spot with a minimal size of 0.05 mm^2^ (corresponding to a ∼175-µm lesion diameter) was considered a GA lesion; smaller lesions were disregarded.^17^ The total lesion size, number of atrophic spots, and the single largest lesion were documented as calculated by the software. For Proxima A images, each GA lesion was individually assessed by two junior graders, with a senior adjudicator when there was disagreement between the junior graders, with a total GA area difference ≥0.25 mm^2^. The adjudication rate, as determined by available data, was 10%. For Proxima B images, GA lesions were individually assessed by a junior and a senior grader, and the average of total lesion size values from the two graders was used as the final value, provided both values were within the predefined tolerance level of ±0.3 mm^2^. Otherwise, a second senior grader determined an additional measurement, and the average of both senior graders was used as the final value.

**Figure 1.**
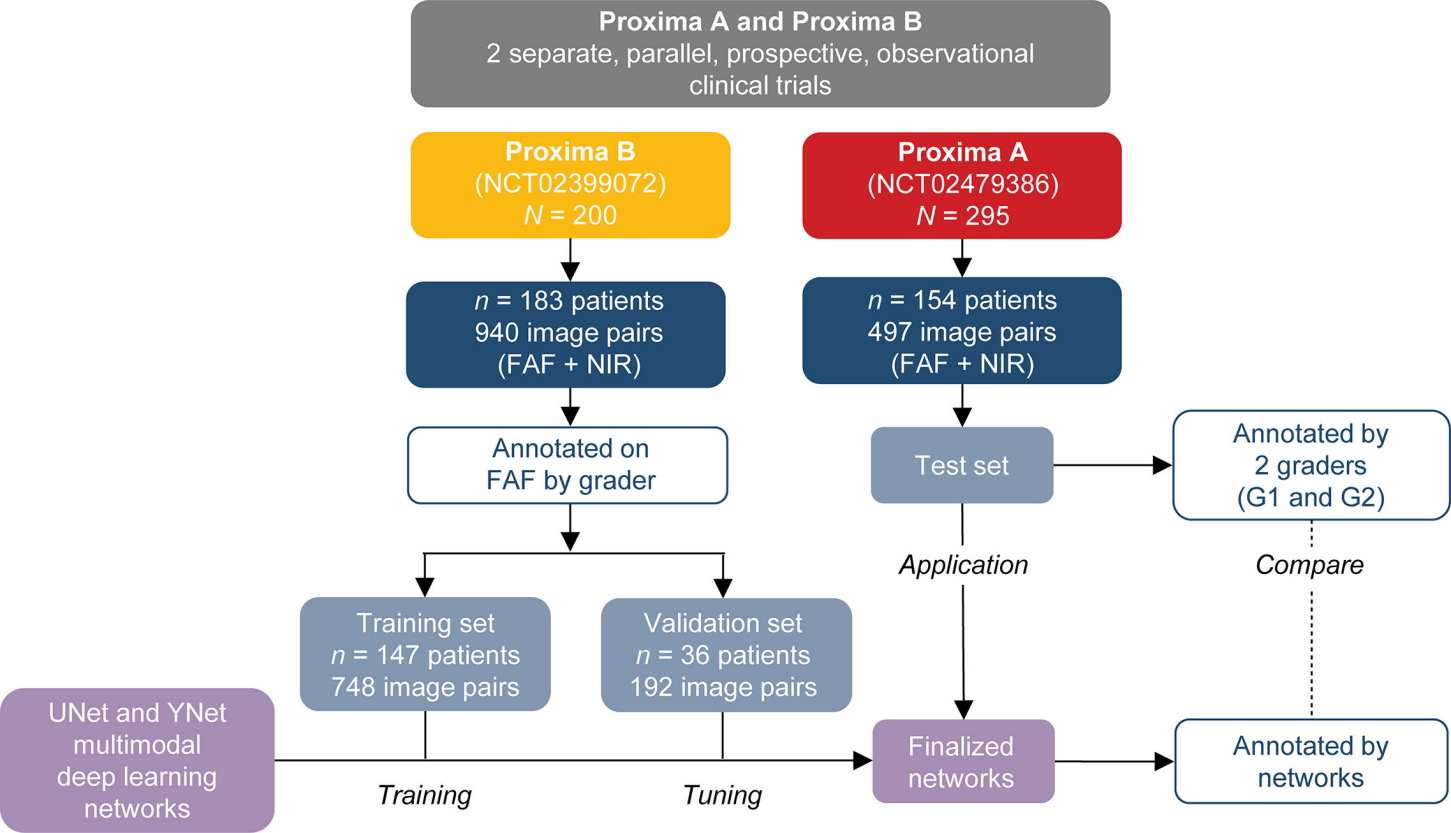
Geographic atrophy (GA) lesion segmentation analysis workflow. FAF, fundus autofluorescence; G1, grader 1; G2, grader 2; NIR, near-infrared reflectance.

In Proxima A, only FAF images with available annotations by both graders (*n* = 185) and, in Proxima B, only FAF images with available annotations by senior grader (*n* = 199) were included and used to create the ground truth. Furthermore, only patients with both FAF and NIR image pairs were included in the analysis; 2 patients in Proxima A and 16 patients in Proxima B did not have corresponding NIR images available and were excluded from this study. Also, the FAF images and corresponding annotations included in the analysis were longitudinally registered by the reading centers using RegionFinder. RegionFinder also has a processing step to improve the contrast and brightness of the images. This study did not incorporate the processing step and used only the original-intensity FAF images generated by the device. Imaging data from Proxima B (940 FAF–NIR image pairs from 183 patients) were split at the patient level into the training set (748 image pairs from 147 patients) and the validation set (192 image pairs from 36 patients; Fig. 1). Imaging data from Proxima A (497 FAF–NIR image pairs from 154 patients) were used as the test data set for evaluating algorithm performance versus human graders (Fig. 1). The number of patients from Proxima A who were included in the current study varied between the screening visit and the subsequent follow-up visits due to some annotations being unavailable at screening or follow-up visits for this study (Table 1).

**Table 1.** Baseline Demographics (Test Data Set From the Proxima A Clinical Trial)

Study Visit	*n*	Female, *n*	Age, Mean (SD), y
Total^a^	154	93	77.75 (7.59)
SCR	89	57	77.46 (7.44)
M6	130	79	77.81 (7.39)
M12	104	61	77.45 (7.66)
M18	54	29	77.87 (7.68)
M24	23	13	79.39 (6.33)
ET	97	56	76.56 (7.29)
SCR and M6^b^	77	50	76.79 (7.32)
SCR and M12^b^	53	33	77.13 (7.33)
SCR and M6 and M12^b^	46	28	76.37 (7.04)

*n* is the number of patients; each patient contributed one eye, and each eye is included at most once at each time point. For age, all ages older than 90 years were treated as 90 years (exact age is unavailable due to anonymization). ET, early termination; G1, grader 1; G2, grader 2; M, month; SCR, screening.

a Total contains all patients included at least once at any time point. SCR through ET are specific time points and refer to the patients used at those time points.

b These rows include those patients with records at all of the listed time points; for example, “SCR and M6” consists of patients with both SCR and M6 images included.

### Model Architectures

FAF and NIR images, resized to 768 × 768 pixels without normalization, were used as inputs for two separate multimodal DL network architectures: UNet and YNet. The outputs from these networks were compared with the ground truth by human graders on FAF images.

The UNet architecture, composed of one encoding and one decoding branch (Supplementary Fig. S1A), is designed to predict and classify each pixel within an image^20^ and is composed of a contractive encoder and an expansive decoder:

$$\begin{array}{ccc} \left(Z,S\right) & = & E\left(\mathrm{concat}\left(\mathrm{FAF},\mathrm{NIR}\right)\right)\\ P & = & D\left(Z,S\right) \end{array}$$

A network diagram of the encoder E is shown in Supplementary Figure S1B (see Supplementary Appendix for model specifications).

The YNet architecture, inspired from a previously published dual-stream model,^21^ contains two encoder branches to encode FAF and NIR images separately and one joint decoder to decode the embeddings of both encoders (Supplementary Fig. S1C). In this architecture, the FAF and NIR images were encoded separately and concatenated before being decoded. The decoding task in this model is expected to benefit from high-resolution representations as the decoder is connected from different encoders.

The reason behind using two encoders was to help the model learn the modality-specific features from FAF and NIR images. This is described as

$$\begin{array}{ccc} \left(Z_{1},S_{1}\right) & = & E_{1}\left(\mathrm{FAF}\right)\\ \left(Z_{2},S_{2}\right) & = & E_{2}\left(\mathrm{NIR}\right)\\ P & = & D\left(\mathrm{concat}\left(Z_{1},Z_{2}\right),S_{1}\right) \end{array}$$

Here, E_1_ and E_2_ were encoders with the same architecture as the encoder of the UNet, except that each took a single-channel image as input. D also had the same architecture as the decoder of the UNet, but its input had twice as many channels. Note that the residual from the FAF encoder was used in the decoder but not the residual from the NIR encoder.

### Model Training and Development

**Figure 2.**
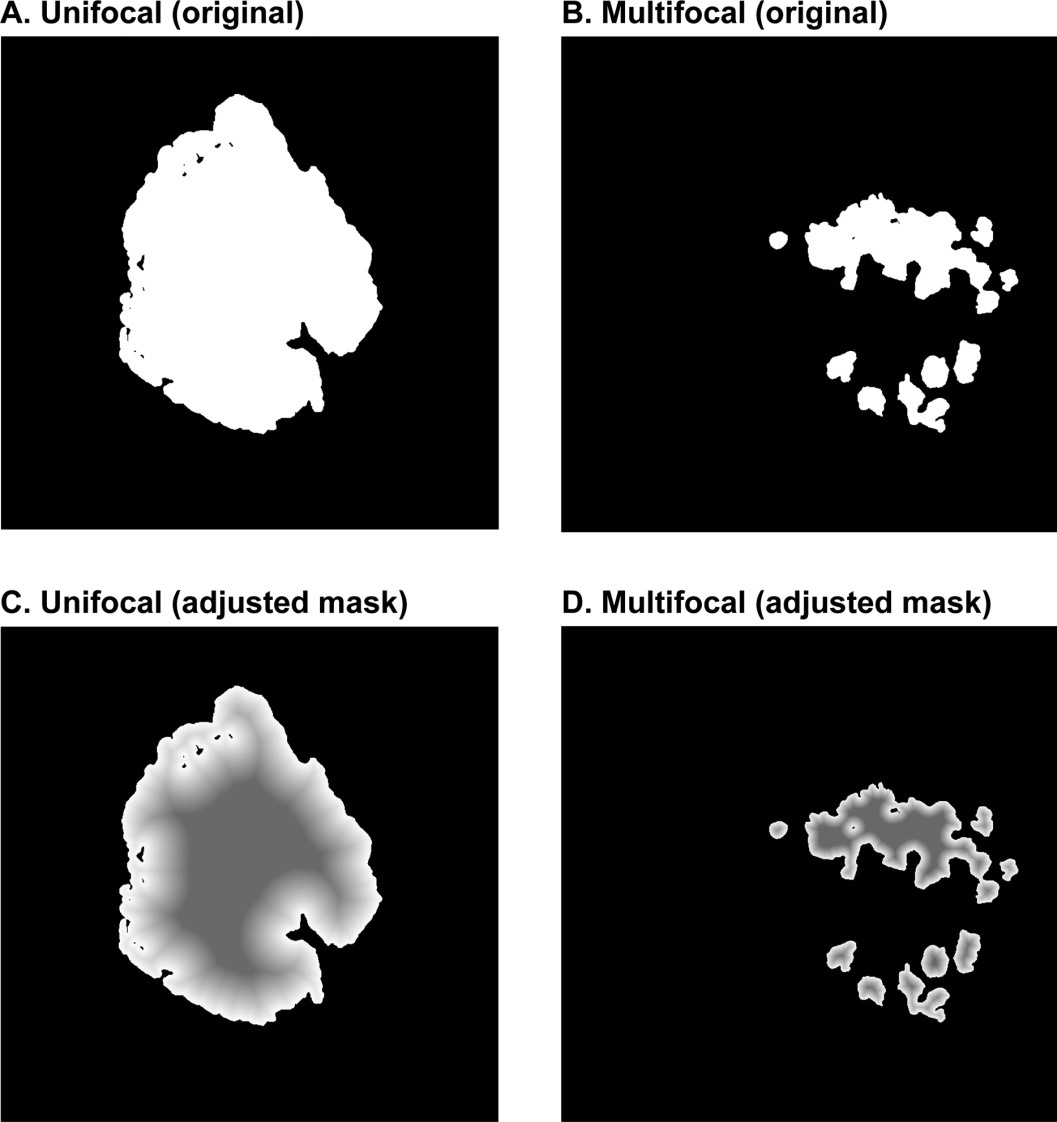
Examples of an original ground-truth GA mask manually annotated by expert grader with values of 0 and 1 of **(A)** a unifocal lesion, **(B)** a multifocal lesion with several small lesions, and **(C, D)** the corresponding modified GA masks with values of 0 where there was no GA lesion and at least 0.5 where there was a GA lesion.

During training, a modified version of the ground-truth masks was used to weight the edges of the lesions more than the interiors (Fig. 2). This follows previous work, which showed that reweighting can help improve the classification accuracy of pixels close to the boundaries.^22^ A modified Dice coefficient was used with the reweighted masks, which put greater emphasis on the borders of the lesion rather than the interior (Fig. 2; Supplementary Appendix). This could be helpful because the algorithm often found the interior of the lesion easier to properly identify, so less emphasis was placed on training it to do so. During validation and testing, original masks were used, and the predictions were assigned a value of 1 when the prediction probability was greater than 0.5 and 0 otherwise. Few other publications have also shown an improvement in model performance using the weighted Dice coefficients.^23^^,^^24^ It is important to note that as the size of the lesion gets smaller, it is possible that the reweighting might not be as effective as in larger-sized lesions.

The described architectures were initialized with random weights. The loss function to be minimized was the Dice loss, defined as 1 minus the Dice coefficient. The hyperparameters tuned during model training were optimizer, learning rate, batch size, learning rate decay, and number of epochs. The validation data set was used to select the hyperparameters. The hyperparameter tuning was performed separately for the UNet and YNet models. The same set of hyperparameters showed the best performance for both models. The selected hyperparameters were Adam optimizer, batch size of 4, and initial learning rate set to 1e−3 and multiplied by 0.1 every 30 epochs for 100 epochs without early stopping. The DL networks trained on the selected hyperparameters were then used to predict the GA lesion segmentation masks on the test set. Python 3.7.7, numpy 1.18.1, PyTorch 1.3.1, and SciPy 1.4.1 were used to train this algorithm. All training was done on Nvidia V100 and P6000 GPUs on an on-premises internal high-performance computing cluster.

### Model Evaluations

The model evaluations were performed on the test set. The cross-sectional performance of the DL networks was assessed using Dice coefficient scores and the Pearson correlation coefficient (*r*). Dice scores measured the similarity of GA segmentations between a DL network (UNet or YNet) and each grader or between the two graders. Generalized Bland–Altman plots with 95% limits of agreement derived from graders were used to assess the agreement of the derived GA lesion areas between algorithms and graders. In addition, the GA lesion areas (in mm^2^) were also correlated between DL networks and graders or between graders, and *r* values were reported together with Passing–Bablok regression analysis. To assess the longitudinal performance, GA lesion enlargement was computed as absolute change (in mm^2^) in GA lesion area from baseline to month 6 and from baseline to month 12, respectively, in the test set. GA lesion enlargement of DL network-predicted segmentation was compared with that of grader annotations, and Bland–Altman plots and scatterplots with *r* values were reported.

## Results

The baseline demographics of patients in Proxima A used in this study to test DL network performance are shown in Table 1.

**Figure 3.**
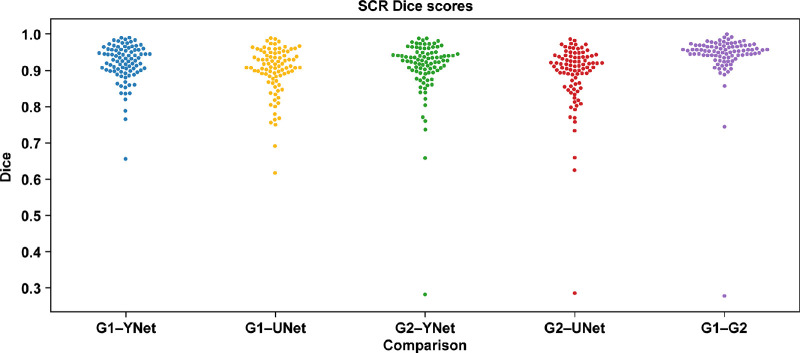
Dice results: similarity of segmentations between the DL networks and the graders or between graders. Results are from the test data set (Proxima A). The comparisons shown are G1–YNet, G1–UNet, G2–YNet, G2–UNet, and G1–G2. The G1–G2 comparison is included to give a reasonable maximum for how well automated algorithms can do.

The UNet model showed a Dice score of 0.95 on the training set and 0.92 on the validation set when compared with the senior grader for all visits. Similarly, the YNet model showed a Dice score of 0.97 on the training set and 0.94 on the validation set. On the test set (Proxima A), the Dice scores for the DL network versus grader comparison for all visits ranged from 0.90 to 0.92 and for the screening visit ranged from 0.89 to 0.92, and the Dice score for the comparison between graders was 0.95 and 0.94 for all visits and the screening visit (Table 2). A summary of the Dice scores for all visits and at each individual visit and a swarmplot of Dice scores at screening visits are shown in Figure 3. These results suggest that the agreement between DL networks and human graders was similar to, or slightly below, the agreement between two graders.

**Table 2.** Dice Results: Dice Scores for all Time Points and at Each Individual Time Point

Study Visit	*n*	G1–YNet	G1–UNet	G2–YNet	G2–UNet	G1–G2
All^a^	497	0.92 (0.09)	0.90 (0.09)	0.91 (0.09)	0.90 (0.09)	0.95 (0.08)
SCR	89	0.92 (0.05)	0.90 (0.07)	0.91 (0.09)	0.89 (0.09)	0.94 (0.08)
M6	130	0.91 (0.10)	0.90 (0.09)	0.92 (0.08)	0.91 (0.07)	0.94 (0.08)
M12	104	0.92 (0.10)	0.90 (0.10)	0.91 (0.11)	0.89 (0.11)	0.95 (0.06)
M18	54	0.93 (0.05)	0.92 (0.05)	0.92 (0.09)	0.91 (0.09)	0.96 (0.08)
M24	23	0.92 (0.05)	0.91 (0.06)	0.89 (0.12)	0.88 (0.11)	0.91 (0.12)
ET	97	0.92 (0.09)	0.90 (0.10)	0.91 (0.08)	0.89 (0.09)	0.95 (0.07)

Results are from test data set (Proxima A). The comparisons we show are YNet–G1, UNet–G1, YNet–G2, UNet–G2, and G1–G2.

a Total number of image pairs.

**Figure 4.**
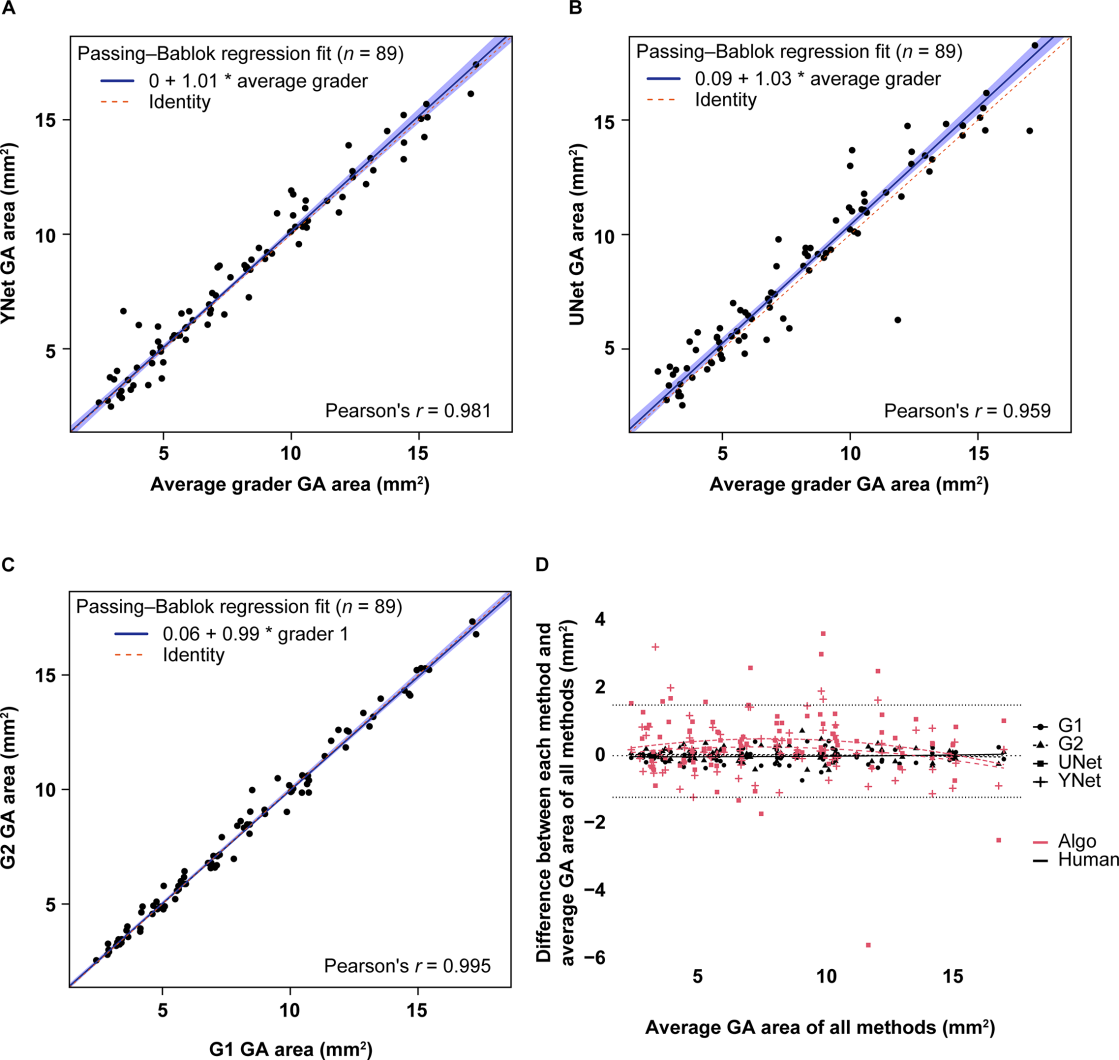
Cross-sectional GA area comparisons at screening (89 image pairs from 89 patients). Results are from the test data set (Proxima A). **(A–C)** A Passing–Bablok regression line is shown between the GA areas derived from two graders. The comparisons shown here are **(A)** YNet versus average grader, **(B)** UNet versus average grader, and **(C)** G1 versus G2. The Pearson correlation coefficient (*r*) is shown for each. **(D)** In the Bland–Altman plot, the x-axis is the average of all graders' areas, and the y-axis is the difference of the individual grader minus the average. A smoothing line (degree 2 polynomial) is included to show general trends. *Dotted lines* represent 95% agreement limits.

In addition, the GA lesion areas (in mm^2^) were also correlated between DL networks and graders or between graders at screening (*n* = 89), and *r* values were reported. The average of the two graders' areas on each image was taken as the standard for grader areas. To set a benchmark for human agreement, the two graders were also compared with each other. Cross-sectional area correlations between YNet and UNet versus the average grader were *r* = 0.981 and *r* = 0.959, respectively (Figs. 4A, 4B)**,** which were similar to the correlation between the two graders (*r* = 0.995) (Fig. 4C)**.** Bland–Altman plots with 95% agreement limits based on the graders only were also used to assess area comparisons (Fig. 4D)**.** Pairwise cross-sectional comparisons for DL network versus each grader are shown in Supplementary Figure S2.

**Figure 5.**
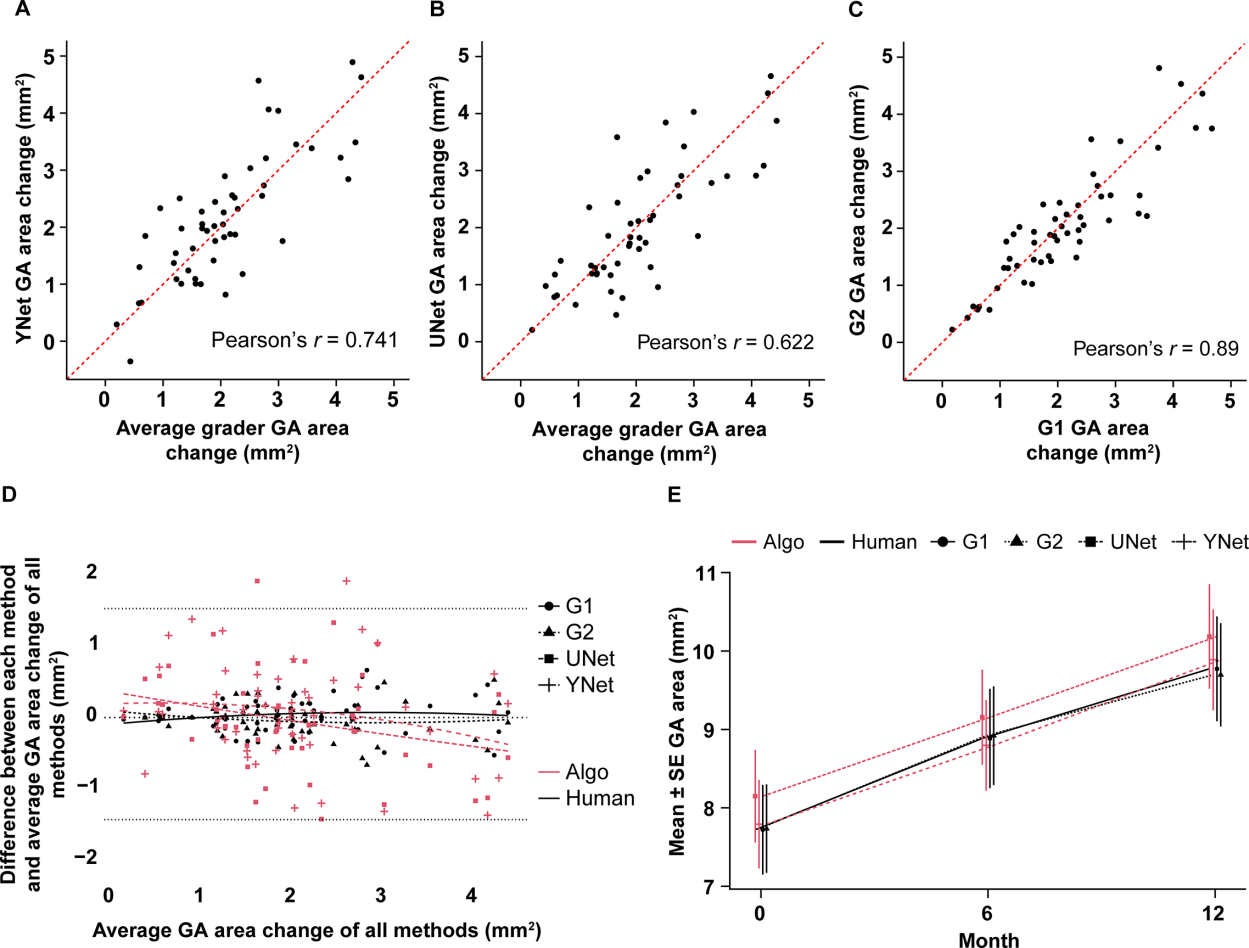
Longitudinal GA area change comparisons from screening to month (M) 12 (*n* = 53). Results are from the test data set (Proxima A). (A–C) A Passing–Bablok regression line is shown between the GA changes derived from two graders. The comparisons shown here are **(A)** YNet versus average grader, **(B)** UNet versus average grader, and **(C)** G1 versus G2. The Pearson correlation coefficient (*r*) is shown for each.^*^ **(D)** In the Bland–Altman plots, the x-axis is the average of all graders' areas, and the y-axis is the difference of the grader minus the average. A smoothing line (degree 2 polynomial) is included to show general trends. **(E)** Area changes from baseline to M6 and baseline to M12 for each grader and network. Mean and SE are shown and graphed.^†^ Note this analysis only uses cases for which all three (baseline, M6, and M12) annotations from both graders were available. ^*^Pearson correlation coefficients shown in figure include outliers. Pearson correlations coefficients after removing any outliers were 0.792 for YNet versus average grader comparison (one outlier removed) and 0.79 for the UNet versus average grader comparison (three outliers removed); these four outliers were removed from the Bland–Altman analysis. ^†^Coefficient of variation between DL network and graders: grader 1: 0.58 (M6) and 0.53 (M12); grader 2: 0.58 (M6) and 0.51 (M12); YNet: 0.8 (M6) and 0.51 (M12); UNet: 1.13 (M6) and 0.6 (M12).

To test the ability of the DL networks to measure area changes by measuring the area of the same eye at different time points, these analyses were also performed on the changes in area from screening to months 6 and 12. Longitudinal area correlations from screening to 12 months (*n* = 53) were lower (*r* = 0.741, *r* = 0.622, and *r* = 0.890, respectively, for YNet versus grader, UNet versus grader, and between graders; Figs. 5A–C), compared with the cross-sectional results. The longitudinal GA area correlations from screening to 6 months (*n* = 77) were even lower (*r* = 0.294, *r* = 0.248, and *r* = 0.686, respectively; Supplementary Figs. S3A–C)**.** Bland–Altman plots (Fig. 5D; Supplementary Fig. S3D) were also used to compare the differences between the GA area changes determined by the grader and the DL network. However, the comparison of measured changes over time (Fig. 5E) demonstrated similar mean change and coefficient of variation (CV) between DL networks and graders, suggesting that although correlation is not high at the individual patient level or over short follow-up periods, the DL networks perform well over time in measuring the endpoint. Pairwise longitudinal comparisons for DL network versus each grader at months 12 and 6 are shown in Supplementary Figure S4 and Figure S5.

Dependence of accuracy of the DL networks on baseline lesion area, focality, and foveal involvement was also assessed on the test data set (Proxima A). In general, there was no strong correlation of algorithm accuracy with any of the clinical factors (Supplementary Figs. S6A–C), indicating that the DL networks work well in those subgroups.

**Figure 6.**
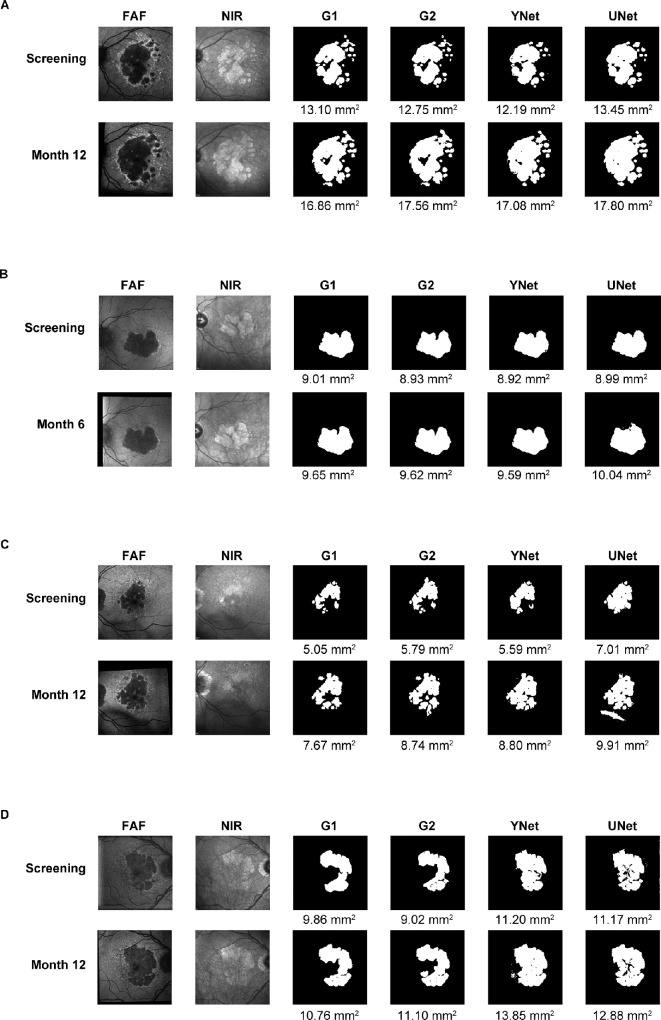
Illustrative examples of good or poor agreement between GA segmentation on FAF performed by expert graders or DL networks, in multifocal or unifocal GA lesions. **(A)** Multifocal lesion and good agreement. **(B)** Unifocal lesion and good agreement. **(C)** Multifocal lesion and poor agreement. **(D)** Unifocal lesion and poor agreement.

Figure 6 shows illustrative examples of good (Figs. 6A, 6B) and poor (Figs. 6C, 6D) agreement between human graders and the DL networks for the GA areas segmented on FAF. The most common area of discrepancy is related to foveal assessment, with the DL networks occasionally identifying the foveal area as part of the GA lesion. The intuition behind using YNet was to help the model learn modality-specific features from FAF and NIR using separate encoders. Certain features are clearly seen in NIR but not in FAF and vice versa (e.g., the fovea is clearly separated from the lesion in NIR but not in FAF). In general, although YNet seems to perform better than UNet at not segmenting the fovea, the differences and any foveal-specific better performance of YNet are minor overall. An example of performance improvement with YNet can be seen in Figure 6C. The “line” seen in the UNet segmentation at month 12 is undoubtedly due to vitreous opacities and not due to GA; however, the graders and YNet correctly judged the line as an artifact and not as a GA lesion. Discrepancies may also be due to the DL networks misinterpreting shadows as lesions or due to poor FAF quality. It must also be noted that although correlation of total GA lesion area is high, the contours can differ greatly in some cases.

## Discussion

In this study, we utilized UNet and YNet architectures on FAF and NIR images for automatic segmentation of GA lesions. State-of-the-art performance was observed for cross-sectional comparisons (*r* > 0.95) between the DL networks and the human graders. We would also like to clarify that the YNet model architecture described in this study is different from the Y-Net described in a previous work.^23^ The Y-Net model developed by Farshad et al.^23^ combines spectral information with spatial information in OCTs using two encoders for improving the segmentation, whereas our work focuses on combining the spatial information from two different imaging modalities using two encoders. Although the networks look similar, the reason for using two encoders is different in both networks.

Previous studies have also evaluated semiautomated or automated image analyses^5^^,^^6^^,^^10^^,^^11^^,^^13^^,^^15^^,^^16^^,^^18^^,^^25^^–^^33^ and reported good algorithm performance between CNN-based networks and graders to measure or segment GA lesions on FAF,^5^^,^^6^^,^^10^^,^^11^^,^^18^^,^^26^^,^^27^^,^^29^^,^^31^ OCT,^13^^,^^16^^,^^28^^,^^30^ or NIR^15^^,^^26^^,^^31^ images. The performance of the models in the present study was comparable to previous publications. A retrospective study on segmentation of 79 FAF–Green images obtained from 62 patients using UNet showed a mean overlap ratio of 92.76% between the manual and DL segmentations.^10^ Another study on 702 FAF images from 51 patients had average training and validation Dice scores of 0.9874 and 0.9779.^11^ A study of 56 SD-OCT scans with GA generated high-quality synthesized FAF images synthetically and proposed a segmentation network that achieved a Dice similarity coefficient 87.2%.^16^ However, most of the publications focus on cross-sectional analyses, which do not necessarily address challenges in charting the progression of GA over time. The present study examined longitudinal correlations in addition to cross-sectional correlations, which may be more relevant to assessing the anatomic primary endpoint in clinical trials, and disease progression in clinical practice. Although the observed longitudinal correlations were lower (*r* = 0.741 for YNet versus grader; *r* = 0.622 for UNet versus grader; CV at month 12 for YNet and UNet were 0.51 and 0.6, respectively) compared with the cross-sectional correlations, population mean and CV were comparable to manual assessment, suggesting that the DL networks performed reasonably well. The current models were tested on observational trial data, and the rate of growth of GA lesions was similar between the graders and the models (Fig. 5E). If the models were applied to interventional trial data, then it is possible that the trial results would not have changed significantly; however, this will need to be confirmed by future studies.

In this study, graders were able to view the previous visit FAF image while annotating the current visit FAF image. This represented an additional quality control step for graders to verify lesions that had a reduced lesion area in follow-up visits; intergrader agreement was also monitored and adjudicated as required. However, the DL networks developed in this study did not have the opportunity to compare the segmentation against imaging data from previous visits (which was done by the graders when performing the manual tracings) and treated each image independently. This is an important aspect to be considered for future algorithm development and can help the models learn the corrections to be applied in case of lesion regression or any other issues. On visually inspecting the cases where the longitudinal performance of the DL networks was not as good as that of human graders, the FAF image quality was poor in screening and/or follow-up visit images (Supplementary Fig. S7).

The ground truth for DL network training was based on annotation of FAF images obtained by trained graders using semiautomated software. The choice of using Proxima B as the training set and Proxima A as the test set was made because developing the DL networks based on more senior grading and testing it on a data set where the graders were of equal experience was more logical than developing the network based on a junior grader and then evaluating it on a data set where a junior and senior grader annotated the lesions. The high agreement between the graders of the test set instills confidence in the quality of the annotations; nonetheless, it is a potential limitation to be addressed in future work with additional validation of the trained DL networks.

No annotations were performed on the NIR images, which were used by the graders as a supportive image modality. The use of NIR images is limited due to insufficient specificity for the GA lesion; while a decrease in FAF is relatively specific to RPE loss, the contrast of highly reflective structures is much lower in GA, and other abnormalities such as hyperpigmentary changes and crystalline deposits not necessarily associated with GA can affect NIR documentation. Human gradings were performed at two different reading centers, in accordance with their respective protocols and quality control practices, which, although similar, could lead to differences in ground-truth imaging grading. Available annotations varied between certain visits (screening, month 6, and month 12) because annotations were not saved and organized as part of the primary clinical trial records, emphasizing the need to prioritize the management of imaging annotations in future studies.

The CNNs described in this study require validation in independent and heterogeneous data sets based on specific use cases for their prospective deployment (e.g., to measure the primary endpoint in future clinical trials or as a potential clinical support tool for patient care). It also remains to be seen whether the AI-based algorithm can be applied to FAF imaging obtained from instruments used in clinical practice globally other than the Spectralis platform used here or to segment atrophic lesions in patients with a diagnosis other than GA secondary to age-related macular degeneration.

In conclusion, this work demonstrates the feasibility of DL-based automatic segmentation of GA lesions in FAF and NIR images from clinical trials in comparison with expert graders. The UNet and YNet DL networks demonstrated good correspondence with experienced graders in cross-sectional GA segmentation using a small data set. Future work will focus on improving the ability of these algorithms to accurately measure longitudinal GA lesion changes. The AI-based algorithms explored in the current study and other recent studies could allow for more quantitative assessment of disease progression and may contribute to new clinical trial endpoints with which to assess the efficacy of medical interventions for GA,^7^ as well as provide valuable information to clinicians and patients about individualized disease prognosis.

## Supplementary Material

Supplement 1
