# Supplementary material for: Geographic Atrophy Segmentation Using Multimodal Deep Learning
Source: Transl Vis Sci Technol. 2023 Jul 10;12(7):10. doi: 10.1167/tvst.12.7.10 (PMC10341294; doi:10.1167/tvst.12.7.10)

## SUPPLEMENTARY APPENDIX

### Model Specifications

Briefly, E alternates convolution blocks and downsampling operations, such that six downsamples were performed in total. Each convolution block consisted of two 3 × 3 convolutions. Each convolution was followed by a GroupNorm[34] and ReLU activation. The downsampling was performed by a 2 × 2 maxpool operation. Z was the final encoded image, and S was the set of partial results of E from before each downsampling step. In the decoder D, we alternated upsampling steps and convolution blocks such that six upsamples were performed in total. The convolution blocks were as in the encoder; the upsampling was done by 2 × 2 transposed convolution. After each upsampling, the same-sized partial result from S was copied in a skip connection and concatenated to the result. After the last convolution block, a 1 × 1 convolution was performed followed by a sigma activation to yield a probability map between 0 and 1.

### Modified Masks and Smoothed Dice Coefficient

The modified masks were defined as follows:

$$mask_{edit} \leftarrow edt\ (mask_{orig})$$

$$mask_{normalized} \leftarrow min\ (mask_{edt}/max(mask_{edt}),\ 0.5)$$

$$mask_{modified} \leftarrow mask_{orig} * (1 - mask_{normalized})$$

Here, $mask_{orig}$ was the original ground truth mask with values 0 and 1, and *edt* was the Euclidean distance transform. All operations other than *edt* and *max* were elementwise operations; *max* returned the maximum value of the input array. The result was a mask

that was 0 where there was no lesion and at least 0.5 where there was a lesion. However, values were higher (up to 1) near the edges of the lesion (Fig. 2).

The smoothed Dice coefficient used was defined as:

$$Dice = \frac{2 * \sum\left(pred * mask_{modified}\right) + \varepsilon}{\sum(pred) + \sum\left(mask_{modified}\right) + \varepsilon}$$

Here, in this case, $\varepsilon = 1$ was used. The gradient of the Dice coefficient with this modification still pushed the algorithm to output the correct 0-1 mask. In other words, the algorithm was pushed to increase probabilities (up to 1) where there was a lesion and decreased them (down to 0) where there was no lesion. However, it put greater emphasis on the borders of the lesion rather than the interior. Note: the epsilon value of one was used to ensure the numerical stability and validity of the smooth Dice score regardless of lesion presence in the original (segmented) image. Provided the ground truth mask has reasonably large positive regions, the value of epsilon generally has negligible effects.

SUPPLEMENTARY FIGURE S1. (A) UNet diagram. (B) Model specification. (C) YNet diagram. FAF, fundus autofluorescence; NIR, near-infrared.

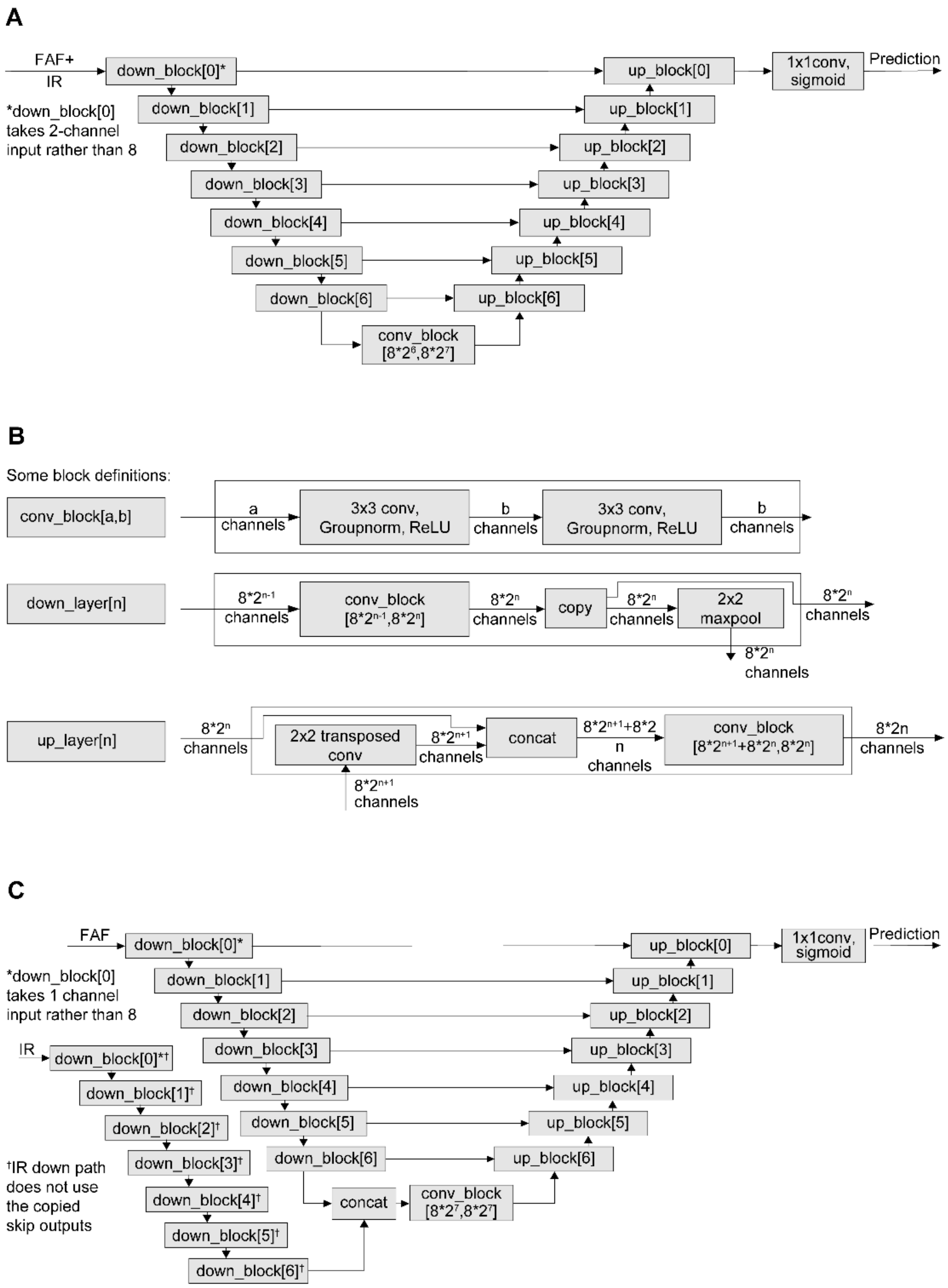

SUPPLEMENTARY FIGURE S2. Pairwise cross-sectional geographic atrophy (GA) area comparisons at screening (89 image pairs from 89 patients). Results are from the test dataset (Proxima A). (A–D) A Passing–Bablok regression line is shown between the GA areas derived from graders. The comparisons shown here are (A) YNet versus grader 1 (G1), (B) Ynet versus grader 2 (G2), (C) UNet versus grader 1, and (D) UNet versus grader 2. The Pearson correlation coefficient (*r*) is shown for each comparison. (E–F) In the Bland–Altman plot, the x-axis is the average of respective graders' areas and the y-axis is the difference of the individual grader minus the average.* A smoothing line (degree 2 polynomial) is included to show general trends. Dotted lines represent 95% agreement limits.

* One outlier was removed from the Bland–Altman analysis.

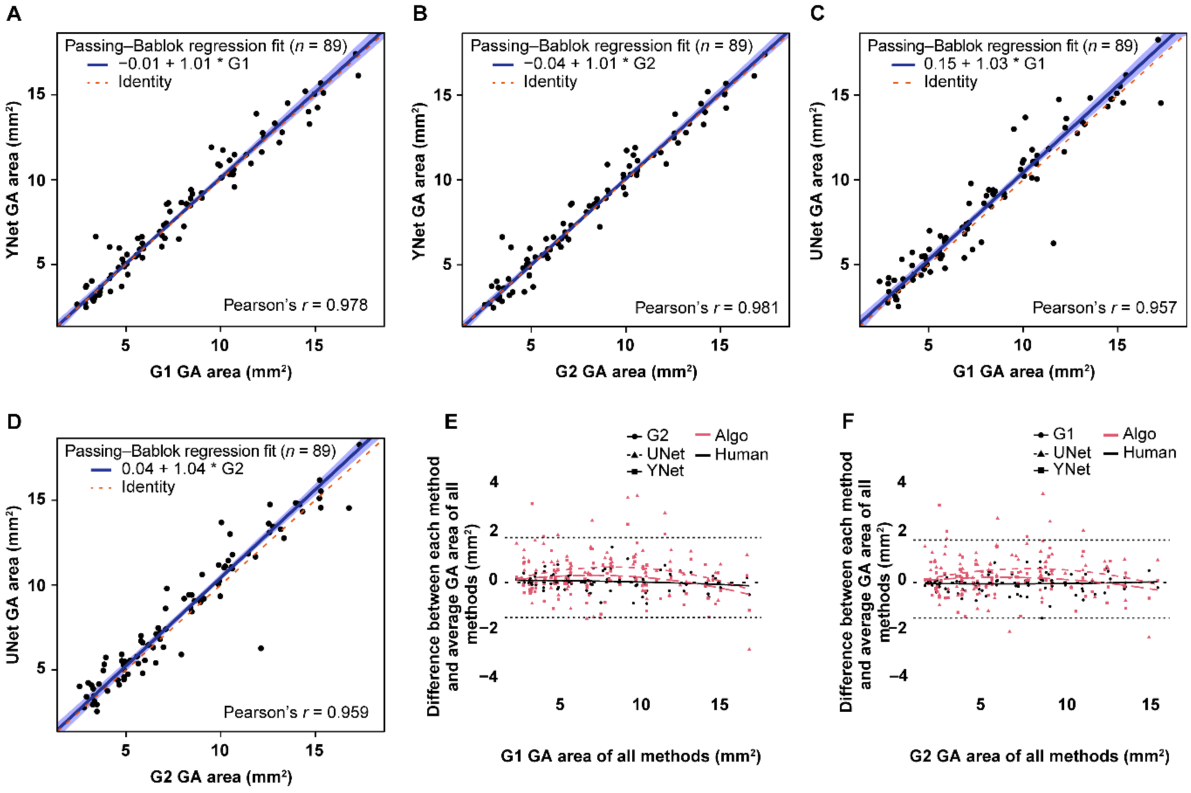

SUPPLEMENTARY FIGURE S3. Longitudinal geographic atrophy (GA) area comparisons from screening to month (M) 6. Results are from test dataset (Proxima A). Patients with screening and month 6 images were included ($n$ = 77). (A–C) A Passing-Bablok regression line is shown between the area changes derived from 2 graders. The comparisons shown here are (A) YNet vs average grader, (B) UNet vs average grader, and (C) grader 1 (G1) versus grader 2 (G2). The Pearson correlation coefficient ($r$) is shown for each.* Note that the areas are annualized, so the numbers shown are double the actual change in area. (D) In the Bland–Altman plots, the x-axis is the average of all graders' areas, and the y-axis is the difference of the individual grader minus the average. A smoothing line (degree 2 polynomial) is included to show general trends

* Pearson correlation coefficients shown in figure include outliers. Pearson correlations coefficients after removing any outliers were: 0.503 for the Ynet vs average grader comparison (2 outliers removed); 0.438 for the UNet vs average grader comparison (3 outliers removed); these 5 outliers were removed from the Bland–Altman analysis.

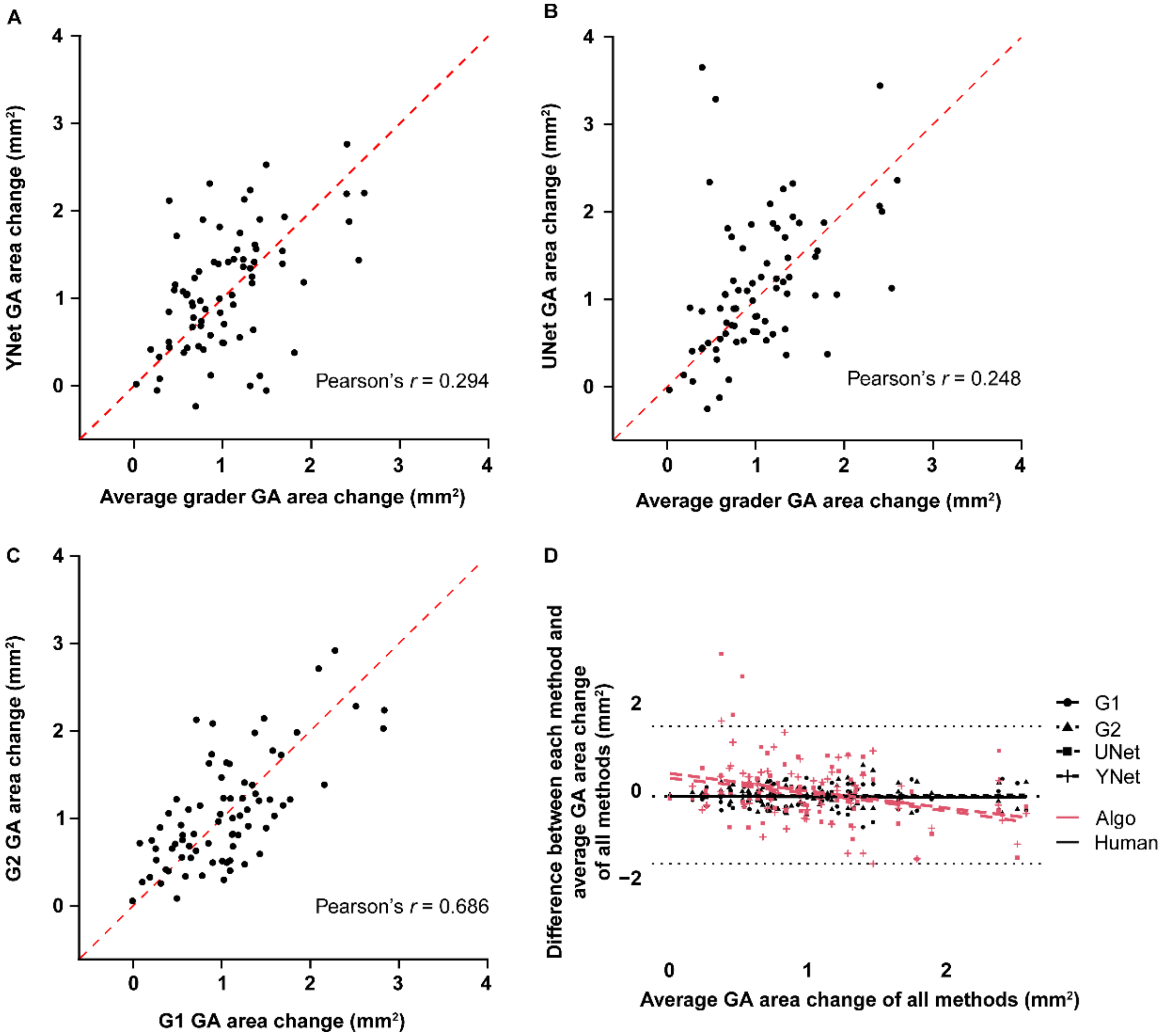
A
YNet GA area change (mm²)
Average grader GA area change (mm²)
Pearson's r = 0.294
B
UNet GA area change (mm²)
Average grader GA area change (mm²)
Pearson's r = 0.248
C
G2 GA area change (mm²)
G1 GA area change (mm²)
Pearson's r = 0.686
D
Difference between each method and average GA area change of all methods (mm²)
Average GA area change of all methods (mm²)
G1
G2
UNet
YNet
Algo
Human

SUPPLEMENTARY FIGURE S4. Pairwise longitudinal geographic atrophy (GA) area change comparisons from screening to month 12. Results are from the test dataset (Proxima A). (A–D) A Passing–Bablok regression line is shown between the GA changes derived from individual grader. The comparisons shown here are (A) YNet versus grader 1 (G1), (B) YNet versus grader 2 (G2), (C) UNet versus grader 1, and (D) UNet versus grader 2. The Pearson correlation coefficient (*r*) is shown for each.* (E-F) In the Bland–Altman plots, the x-axis is the average of the individual graders' areas, and the y-axis is the difference of the grader minus the average. A smoothing line (degree 2 polynomial) is included to show general trends. Dotted lines represent 95% agreement limits.

* Pearson correlation coefficients shown in figure include outliers. Pearson correlations coefficients after removing any outliers were: 0.803 for the Ynet versus grader 1 comparison (1 outlier removed); 0.741 for the Ynet versus grader 2 comparison (1 outlier removed); 0.793 for the UNet versus grader 1 comparison (3 outliers removed) and 0.749 for the UNet versus grader 2 comparison (3 outliers removed); these 4 outliers were removed from the Bland–Altman analysis.

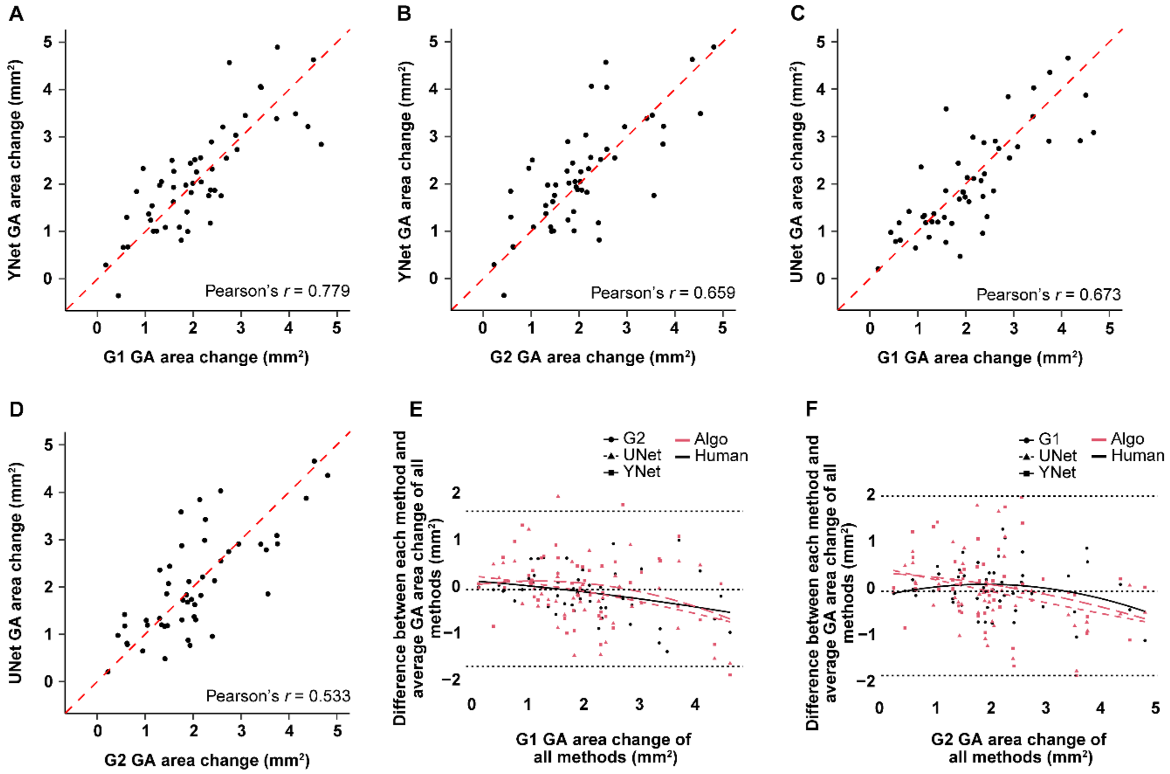
A
YNet GA area change (mm²)
G1 GA area change (mm²)
Pearson's r = 0.779
B
YNet GA area change (mm²)
G2 GA area change (mm²)
Pearson's r = 0.659
C
UNet GA area change (mm²)
G1 GA area change (mm²)
Pearson's r = 0.673
D
UNet GA area change (mm²)
G2 GA area change (mm²)
Pearson's r = 0.533
E
G2
UNet
YNet
Algo
Human
Difference between each method and average GA area change of all methods (mm²)
G1 GA area change of all methods (mm²)
F
G1
UNet
YNet
Algo
Human
Difference between each method and average GA area change of all methods (mm²)
G2 GA area change of all methods (mm²)

Supplementary Figure S5. Pairwise longitudinal geographic atrophy (GA) area change comparisons from screening to month 6. Results are from the test dataset (Proxima A). (A–D) A Passing–Bablok regression line is shown between the GA changes derived from individual grader. The comparisons shown here are (A) YNet versus grader 1 (G1), (B) YNet versus grader 2 (G2), (C) UNet versus grader 1, and (D) UNet versus grader 2. The Pearson correlation coefficient (*r*) is shown for each.* (E–F) In the Bland–Altman plots, the x-axes are the average of the individual graders' areas, and the y-axes are the difference of the grader minus the average. Smoothing lines (degree 2 polynomial) are included to show general trends. Dotted lines represent 95% agreement limits.

* Pearson correlation coefficients shown in figure include outliers. Pearson correlations coefficients after removing any outliers were: 0.0.349 for the Ynet versus grader 1 comparison (2 outliers removed); 0.57 for the YNet versus grader 2 comparison (2 outliers removed); 0.314 for the UNet versus grader 1 comparison (3 outliers removed) and 0.491 for the UNet versus grader 2 comparison (3 outliers removed); these 5 outliers were removed from the Bland–Altman analysis.

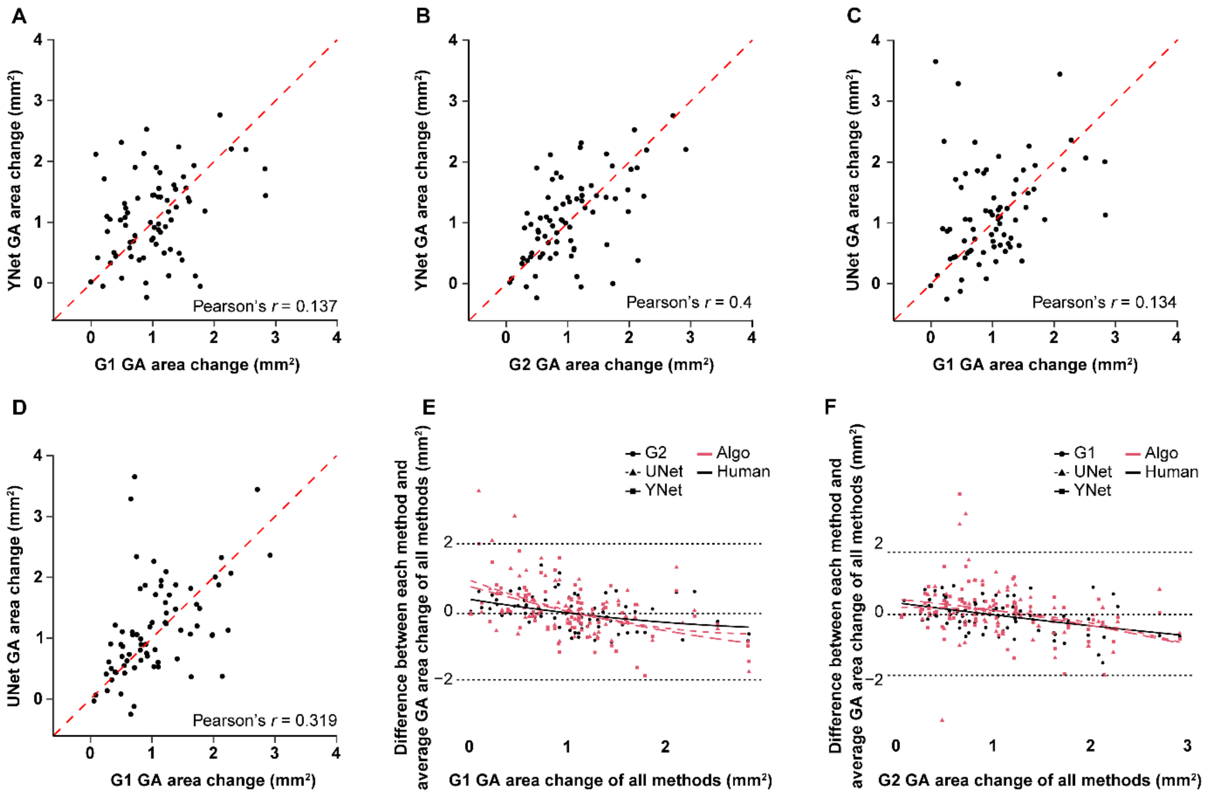
A
YNet GA area change (mm²)
G1 GA area change (mm²)
Pearson's r = 0.137
B
YNet GA area change (mm²)
G2 GA area change (mm²)
Pearson's r = 0.4
C
UNet GA area change (mm²)
G1 GA area change (mm²)
Pearson's r = 0.134
D
UNet GA area change (mm²)
G1 GA area change (mm²)
Pearson's r = 0.319
E
Difference between each method and average GA area change of all methods (mm²)
G1 GA area change of all methods (mm²)
G2
UNet
YNet
Algo
Human
F
Difference between each method and average GA area change of all methods (mm²)
G2 GA area change of all methods (mm²)
G1
UNet
YNet
Algo
Human

SUPPLEMENTARY FIGURE S6. (A) Variance with baseline geographic atrophy (GA) lesion area: discrepancy of the GA area measured by the algorithms (or area change) versus GA area measured by a grader (change) as a function of baseline GA area (as measured by the grader). (B) Variance with baseline focality: discrepancy of the GA area measured by the algorithms (or area change) versus the GA area measured by a grader (change) in unifocal and multifocal lesions.* (C) Variance with baseline foveal involvement: discrepancy of the GA area measured by the algorithms (or area change) versus the GA area measured by graders (change) in subfoveal and nonsubfoveal lesions.

Results are from the test dataset (Proxima A). Screening (SCR): $n = 89$; SCR to month (M) 6: $n = 77$; SCR to M12: $n = 53$. Only YNet vs average grader are shown. *Average grader is not used here because focality cannot be defined in the average grader.

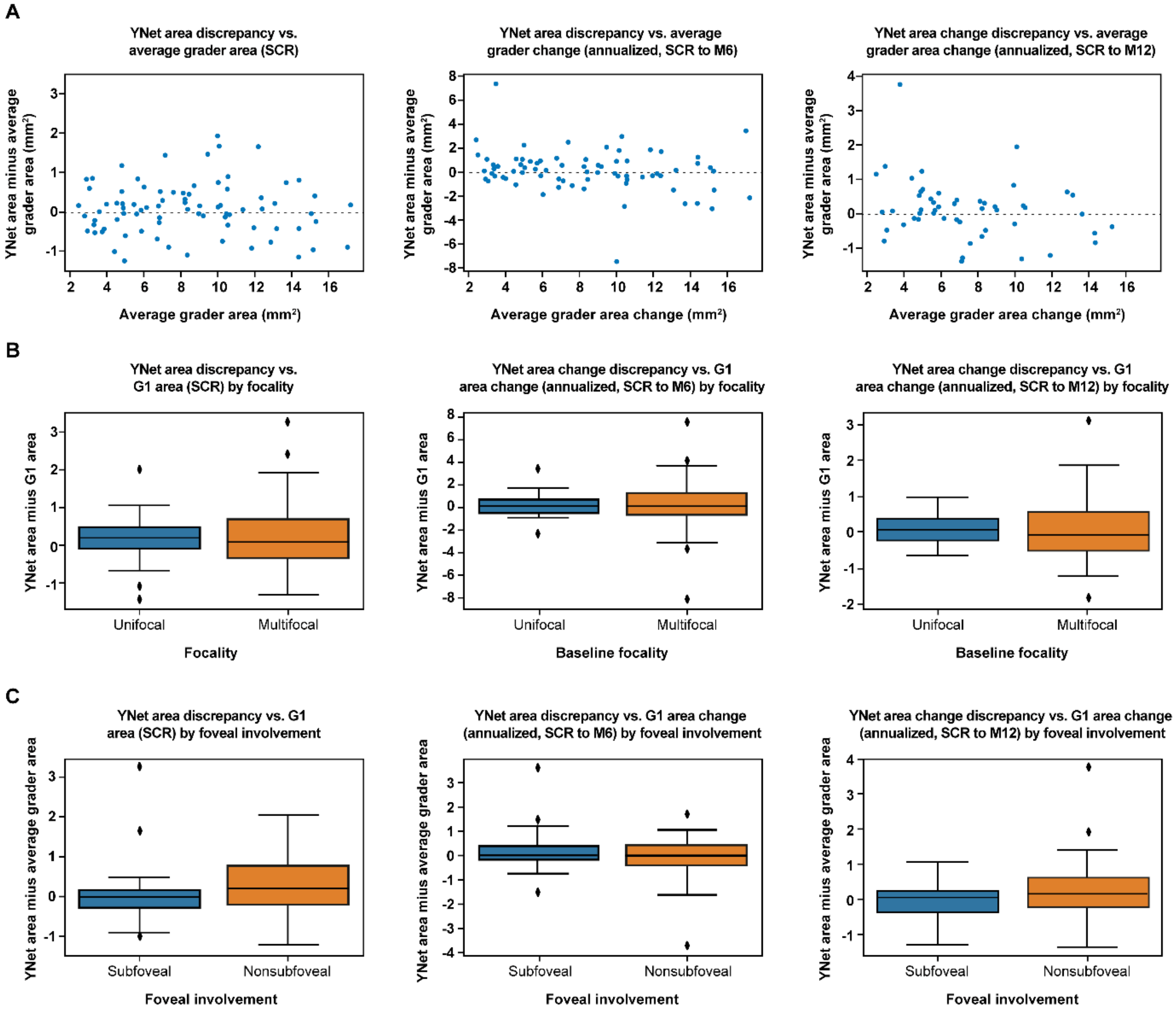
A
YNet area discrepancy vs.
average grader area (SCR)
YNet area minus average grader area (mm²)
Average grader area (mm²)
YNet area discrepancy vs. average
grader change (annualized, SCR to M6)
YNet area minus average grader area (mm²)
Average grader area change (mm²)
YNet area change discrepancy vs. average
grader area change (annualized, SCR to M12)
YNet area minus average grader area (mm²)
Average grader area change (mm²)
B
YNet area discrepancy vs.
G1 area (SCR) by focality
YNet area mius G1 area
Unifocal
Multifocal
Focality
YNet area change discrepancy vs. G1
area change (annualized, SCR to M6) by focality
YNet area mius G1 area
Unifocal
Multifocal
Baseline focality
YNet area change discrepancy vs. G1
area change (annualized, SCR to M12) by focality
YNet area mius G1 area
Unifocal
Multifocal
Baseline focality
C
YNet area discrepancy vs. G1
area (SCR) by foveal involvement
YNet area mius average grader area
Subfoveal
Nonsubfoveal
Foveal involvement
YNet area discrepancy vs. G1 area change
(annualized, SCR to M6) by foveal involvement
YNet area mius average grader area
Subfoveal
Nonsubfoveal
Foveal involvement
YNet area change discrepancy vs. G1 area change
(annualized, SCR to M12) by foveal involvement
YNet area mius average grader area
Subfoveal
Nonsubfoveal
Foveal involvement

Supplementary Figure S7. Examples of poor fundus autofluorescence image quality that were found to affect the longitudinal performance of deep learning networks (A) at screening and at the 12-month follow-up visit and (B) at screening and at the 6-month follow-up visit.

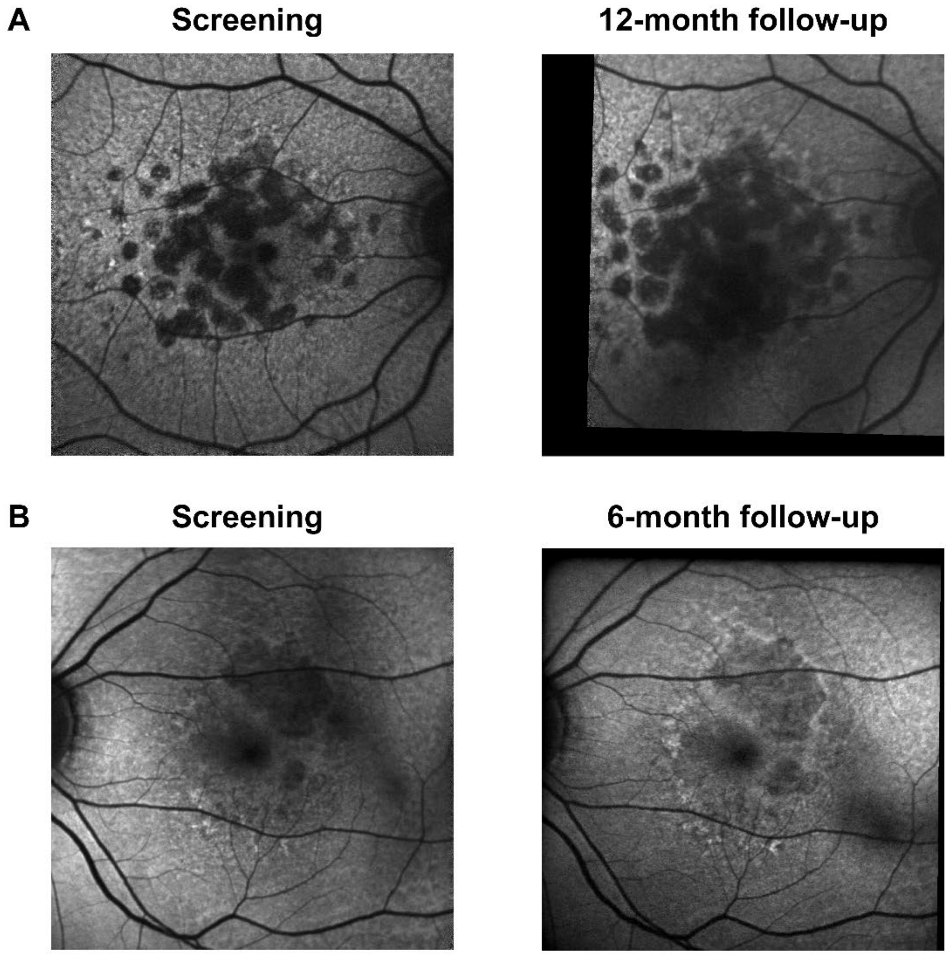

Supplement: Supplement 1 [file tvst-12-7-10_s001.pdf]
